# Significant correlations between blood lipids, cytokines, and C-reactive protein in healthy humans

**DOI:** 10.1186/s12944-026-02939-w

**Published:** 2026-04-09

**Authors:** Anders Larsson, Lars B Eriksson, Mats Eriksson

**Affiliations:** 1 Department of Medical Sciences, Uppsala University, Uppsala University Hospital, Uppsala, SE-751 85 Sweden; 2 Department of Surgical Sciences, Section of Section of Anaesthesiology and Intensive Care Medicine, Uppsala University, Uppsala University Hospital, Akademiska sjukhuset, Entrance 70, 2nd Floor, Uppsala, SE-751 85 Sweden; 3 NOVA Medical School, New University of Lisbon, Lisbon, 1099-085 Portugal

**Keywords:** hsCRP, Blood cells, Cardiovascular, Cytokine, Inflammation, Lipid

## Abstract

**Background:**

Chronic low-grade inflammation plays a central role in cardiometabolic disease, yet the associations between lipid metabolism and inflammatory biomarkers in generally healthy individuals remain incompletely understood. This study aimed to investigate the relationship between blood lipids, high-sensitivity C-reactive protein (hsCRP), and a broad panel of inflammatory cytokines in a healthy adult population.

**Methods:**

A total of 165 healthy participants aged 18–44 years were recruited at the Falun County Hospital, Sweden. Blood samples were analyzed for a full lipid profile, blood counts, cytokines, and hsCRP. Plasma inflammatory protein levels were quantified using the Olink Proseek Multiplex Inflammation panel, including 92 cytokines. Statistical analysis included Spearman rank correlations and multiple testing correction using the Benjamini–Hochberg false discovery rate (FDR < 0.10).

**Results:**

hsCRP showed significant correlations with several lipid parameters, particularly remnants, triglycerides, apolipoprotein B (ApoB), and non-HDL cholesterol, as well as with BMI and specific leukocyte counts. Additionally, hsCRP was significantly associated with multiple cytokines, including IL-6, TNF, IL-10, and CXCL10, highlighting a complex pro- and anti-inflammatory milieu.

**Conclusions:**

This study demonstrates correlations between hsCRP, lipid-related biomarkers, and inflammatory cytokines in healthy adults, underscoring the interplay between lipid metabolism and subclinical inflammation. The significant correlations between hsCRP and remnants, ApoB, and cytokines such as IL-6 support the role of these factors as early indicators of cardiometabolic risk, even in the absence of overt disease.

**Supplementary Information:**

The online version contains supplementary material available at 10.1186/s12944-026-02939-w.

## Introduction

 C-reactive protein (CRP) is a pentameric acute-phase protein primarily produced by the liver in response to inflammation and infection [1–3]. Its synthesis is induced by pro-inflammatory cytokines, especially interleukin-6 (IL-6), and to a lesser extent by tumor necrosis factor alpha (TNF-α), and interleukin-1 beta (IL-1β) [3]. CRP is a rapid, sensitive, and widely available biomarker for detecting and monitoring inflammation and infections. High CRP levels suggest bacterial infections, while mildly elevated levels may indicate viral infections or chronic inflammation [4].

Low-grade CRP elevation is associated with cardiovascular risk, and high-sensitivity CRP (hsCRP) is used in cardiovascular disease (CVD) risk stratification. CRP and blood lipids interact in complex ways as risk factors for CVD, and their combined assessment can enhance risk stratification [5, 6].

Adipose-derived cytokines play an important role in mediating the metabolic consequences of obesity and excess body fat. The cytokines produced result in increased levels of CRP. Thus, there is a clear association between the amount of adipose tissue and CRP synthesis by the liver. Diet-induced weight loss is associated with reduced CRP levels. Obesity and increased BMI are risk factors for CVD [7].

Not only the amount of fatty tissue but also blood lipid levels are associated with increased CVD risk. CRP and blood lipids, such as LDL (low-density lipoprotein) cholesterol (LDLC), triglycerides (TG), non-HDL cholesterol (HDLC), and remnants, are independent predictors of cardiovascular risk. Blood lipids, particularly elevated LDLC and remnant lipoproteins, contribute directly to atherogenesis through cholesterol deposition in arterial walls. When elevated together, CRP and lipid markers indicate a combination of atherogenic burden and inflammatory activity, both of which are central to plaque development and rupture. Triglycerides and remnants are pro-inflammatory, triggering endothelial activation and cytokine release. CRP may promote monocyte recruitment and foam cell formation, creating a feedback loop that drives both inflammation and lipid-driven atherosclerosis [8].

Chronic low-grade inflammation, reflected by hsCRP, may enhance oxidation of LDL particles, triggering foam cell formation and endothelial dysfunction. The inflammation leads to increased endothelial permeability, facilitating lipid infiltration into the vessel wall.

Studies such as the JUPITER trial, have shown that individuals with normal LDLC but elevated CRP still benefit from lipid lowering therapy by statins, suggesting CRP captures inflammatory risk not evident from lipids alone [9, 10]. These findings further support the inflammatory hypothesis of atherosclerosis, as anti-inflammatory therapy significantly reduced recurrent cardiovascular events independently of lipid lowering [11].

These biomarkers are well studied in established disease, but their interrelationships in apparently healthy populations remain insufficiently characterized.

While cardiovascular screening is well established, hsCRP appears underutilized in clinical practice [12]. This study aimed to examine correlations between blood lipids and hsCRP in an apparently healthy cohort, emphasizing an alternative angle on the potential role of inflammatory cytokines, chemokines, growth factors (CCGFs).

## Materials and methods

### Study population

This is a cross-sectional study examining potential correlations between lipid markers, hsCRP, and CCGFs in essentially healthy, volunteer participants without any known cardiac disease, including hypertension and blood lipid disturbances, recruited at the Oral and Maxillofacial Surgery Clinic at Falun County Hospital, Sweden by LBE for mandibular third molar removal. Exclusion criteria: Regular medication with analgesics, hypnotics, thyroid hormones, psychoactive drugs or diagnosed with clinically significant psychiatric disorder, epilepsy, hyperthyroidism, myasthenia gravis, glaucoma, verified sleep apnea, diabetes, porphyria, pregnancy, breast-feeding, blood transmitted infections or if they had a known hypersensitivity to midazolam, ketamine, ibuprofen or local anesthetics. Potential participants were excluded from the study if informed oral and written consent was not obtained [13, 14].

### Blood sampling and analyses

Venous blood samples were obtained from fasting patients via a peripheral venous catheter, with the patients in the supine position, shortly before the onset of the surgical procedure. All blood samples (EDTA) were centrifuged, and plasma was transferred to new tubes. Plasma samples were frozen and stored at − 80 °C until analysis. The samples were thawed, and blood lipids (Apolipoprotein A1, Apolipoprotein B, total cholesterol, HDL cholesterol, and Triglycerides) were analyzed. Complete blood counts and hsCRP were analyzed at the clinical laboratory at Falun Hospital, using Siemens Atellica Solution CH930. Both the laboratories in Falun and Uppsala are accredited according to 15,189 and are participating in external quality assurance programs organized by the Swedish external quality assurance organization Equalis (Uppsala, Sweden).

Total coefficient of variation for the methods used: CRP 1.4% at 2.6 mg/L and 1% at 22 mg/L; Triglycerides 1.9% at 0.8 mmol/L and 1.1% at 2.2 mmol/L; Cholesterol 1% at 3.3 mmol/L and 1% at 5.7 mmol/L; HDLC 2.6% at 0.9 mmol/L and 1% at 2.2 mmol/L.

LDLC was calculated using the Friedewald’s equation [15]. Non-HDLC (Non-High-density lipoprotein cholesterol) and remnants [Non-HDLC – Low-density lipoprotein cholesterol (LDLC)] were derived from the other blood lipid biomarkers.

### Proximity Extension Assay (PEA)

Inflammatory protein levels were measured using the Proseek Multiplex Inflammation kit (Olink Bioscience, Uppsala, Sweden) [16]. The PEA has been shown to reliably reflect protein plasma levels, as compared to conventional assays [17]. A 1 µL aliquot of plasma was incubated overnight at 8 °C with 3 µL of an incubation mix containing two specific DNA-labeled antibodies (probes). After incubation, 96 µL of extension mix, including the enzyme and PCR reagents, was added. Samples were incubated at room temperature for five minutes, followed by 17 cycles of DNA amplification in a thermal cycler.

The 96.96 Dynamic Array IFC (Fluidigm, USA) was prepared per manufacturer instructions. On one side of the array, 2.8 µL of the sample-probe mixture was combined with 7.2 µL of detection mix, and 5 µL was loaded. Unique primer pairs for each cytokine were added to the other side. The array was then processed using the Fluidigm Biomark system. The kit analyzed 92 biomarkers.

The assay expresses protein concentration values on a log₂ scale, which normalizes data distribution, and facilitates biological interpretation of fold changes.

The 92 proteins determined by this assay are displayed with corresponding, UniprotIDs and gene symbols in Supplementary Table 1.

### String images

Interactions among cytokines, chemokines, and growth factors, together with their concentration patterns, were visualized using images generated from the STRING database [18]. Edge weights in STRING were calculated based on the associations identified in this study. Protein names were included to construct interaction networks, with edge thickness representing confidence scores and thus the likelihood of true interactions according to STRING’s integrated evidence. The images were exported at high resolution for figure preparation, enabling simultaneous visualization of quantitative concentration data and qualitative interaction patterns.

### Statistical analysis

Coefficients of correlation were evaluated using Spearman’s rank correlation analysis performed in Statistica (StatSoft, Version 14; Tulsa, OK, USA). Measurements exceeding the upper or lower limits of the standard curve were set to the corresponding highest or lowest standard values. To account for the elevated risk of type I errors arising from multiple comparisons, *p*-values were corrected for multiple testing using the false discovery rate (FDR) method according to Benjamini–Hochberg. An adjusted *p*-value < 0.10, reflecting a maximum expected false discovery rate of 10%, was regarded as statistically significant [19].

Differences in laboratory variables between sexes were assessed using the Mann–Whitney U test. The null hypothesis assumed no sex differences in the analytes.

Correlations were expressed according to Schober et al. [20].

### Ethical considerations

The study adhered to the principles of the Declaration of Helsinki [21] and was approved by the Swedish Ethical Review Authority (approval number: 2015/378). It was registered in the EU Clinical Trials Register (EudraCT No: 2014-004235-39) and on ClinicalTrials.gov (ID: NCT04459377). All participants provided both verbal and written informed consent.

## Results

Clinical, demographic, and laboratory characteristics of the cohort (*n* = 165; 53♂) are presented in Table 1.

**Table 1:** Body mass index (BMI); Apolipoprotein A1 (ApoA1); Apolipoprotein B (ApoB); Total cholesterol (Tot. Chol.); High density lipoprotein cholesterol (HDL Chol.); Non-high density lipoprotein cholesterol (Non HDLC); Low density lipoprotein cholesterol (LDLC); Hemoglobin (Hb); White blood cells (WBC); High sensitivity CRP (hsCRP); Platelets (PLT); Albumin (ALB); Creatinine (Crea)

		Median	Lower Quartile	Upper Quartile
Age	year	29	23	35
Weight	kg	73	64.9	82.1
BMI	kg/m^2^	22	15.3	40.7
ApoA1	g/L	1.59	1.48	1.73
ApoB	g/L	0.87	0.76	1.01
Tot. Chol.	mmol/L	4.57	4.11	5.1
HDLC	mmol/L	1.28	1.14	1.5
TG	mmol/L	0.81	0.65	1.1
Non-HDLC	mmol/L	3.26	2.78	3.73
LDLC	mmol/L	2.80	2.37	3.31
Remnants	mmol/L	0.36	0.29	0.5
Hb	g/L	134	129	145
WBC	x10^9^/L	5.5	4.6	6.7
Neutrophil cells*	x10^9^/L	4.2	3.2	5.2
hsCRP**	mg/L	0.7	0.3	1.7
PLT	x10^9^/L	239	207	273
ALB	g/L	42	40	44
Crea	µmol/L	67	59	76

*Denotes 162 subjects

** Denotes 161 subjects

Significant intercorrelations among demographic variables, as well as correlations between demographic variables and laboratory analytes, are shown in Supplementary Table 2.

Significant sex differences in analytes within our cohort are shown in Table 2.

**Table 2:** Sex differences in Apolipoprotein A1 (ApoA1); Apolipoprotein B (ApoB); High density lipoprotein cholesterol (HDLC); Triglycerides (Triglyc.); Non- High density lipoprotein cholesterol (HDLC); Remnants (Non-HDLC – LDLC; mmol/L)

**Analyte**	**Z**	***p*** **-value**
ApoA1 (g/L)*	2.42884	0.015148
ApoB (g/L)*	-2.28227	0.022474
HDLC (mmol/L)*	2.66789	0.007633
Triglyc. (mmol/L)*	-2.48816	0.012841
Non-HDLC (mmol/L)*	-2.21422	0.026814
Remnants (mmol/L)*	-2.48816	0.012841
ALT*	-3.27684	0.001050
uPA**	-3.75509	0.000173
IL-17 C**	-4.22624	0.000024
MCP-1**	-3.54413	0.000394
IL18**	-2.17289	0.029789
SLAMF1**	-2.03928	0.041423
CCL11**	-3.83244	0.000127
IL-15RA**	-2.11663	0.034292
IL-18R1**	-2.94993	0.003179
PD-L1**	-2.27134	0.023127
TRANCE**	-2.97102	0.002968
HGF**	-2.82687	0.004701
MMP-10**	-2.36979	0.017799
Flt3L**	2.35221	0.018663
DNER**	-4.09966	0.000041
EN-RAGE**	-3.43865	0.000585
FGF-19**	-1.98654	0.046974
CASP-8**	-2.77061	0.005595
CCL25**	-3.19605	0.001393
TNFRSF9**	-3.32614	0.000881
TWEAK**	-3.71993	0.000199
ADA**	-3.17143	0.001517
CSF-1**	1.97248	0.048556

* *N* = 112

** *N* = 111

### Correlations between blood lipids and hsCRP

Remnants, triglycerides, Apolipoprotein B, Non-HDLC, and total cholesterol all showed significant weak positive correlations with CRP. The correlations of remnants and triglycerides, respectively, with hsCRP were also weak (Table 3).

**Table 3:** Significant correlations between blood lipids and hsCRP in descending order in 161 subjects. Remnants (Non-HDLC – LDLC; mmol/L); [High-density lipoprotein cholesterol (HDLC); (mmol/L)], Low-density lipoprotein cholesterol (LDLC)

Blood lipid	Spearman	t(N-2)	*p*-value	Benjamini-Hochberg *p*-values
Remnants (mmol/L)	0.256662	3.3486	0.001014	0.01
Triglycerides (mmol/L)	0.256601	3.3477	0.001017	0.01
Apolipoprotein B (g/L)	0.232911	3.0200	0.002946	0.02269
Non-HDLC (mmol/L)	0.210536	2.7156	0.007347	0.03211
Total cholesterol (mmol/L)	0.185725	2.3834	0.018335	0.05547

In contrast, Apolipoprotein A1 and HDL-cholesterol were not associated with hsCRP.

### Correlations between CRP and cytokine levels

Twenty-nine out of 92 CCGFs displayed significant correlations with hsCRP after Benjamini-Hochberg adjustment for multiple comparisons. There was a moderate correlation between IL6 and hsCRP while the other cytokines showed weak correlations (Table 4).

**Table 4:** Significant correlations between hsCRP and cytokines in descending order in 160 subjects

Cytokine	Spearman	t(N-2)	*p*-value	Benjamini-Hochberg *P*-values
IL6	0.479035	6.85965	0.000000	7.3721E-08
CSF-1	0.349907	4.69506	0.000006	0.00143509
CCL19	0.277311	3.62803	0.000385	0.01477658
CCL3	0.274639	3.59021	0.000441	0.01571777
CDCP1	0.263432	3.43253	0.000763	0.02383509
TNF	0.259653	3.37971	0.000914	0.0260935
AXIN1	0.230420	2.97642	0.003375	0.04886792
MCP-3	0.223916	2.88791	0.004422	0.05663816
OSM	0.222963	2.87498	0.004598	0.05743131
TNFSF14	0.219944	2.83406	0.005196	0.06015241
SIRT2	0.219745	2.83135	0.005238	0.06015241
IL10*	0.213969	2.74459	0.006765	0.06430232
IL-18R1	0.203467	2.61218	0.009864	0.08077448
ADA	0.200998	2.57914	0.010816	0.08312048
STAMBP	0.198773	2.54940	0.011743	0.08500843
VEGFA	0.196862	2.52390	0.012593	0.08561537
CCL20	0.194378	2.49080	0.013779	0.08642647
NT-3	0.193755	2.48250	0.014092	0.08642647
IL-10RB	0.191233	2.44895	0.015420	0.08642647
TWEAK	-0.195931	-2.51150	0.013027	0.08561537
CX3CL1	-0.201393	-2.58443	0.010659	0.08312048
FGF-19	-0.202415	-2.59809	0.010261	0.08247931
CCL11	-0.248113	-3.21939	0.001559	0.0313387
SCF	-0.258039	-3.35720	0.000987	0.02663555
LIF-R	-0.277757	-3.63436	0.000376	0.01477658

* Denotes 159 subjects

**Fig. 1:**
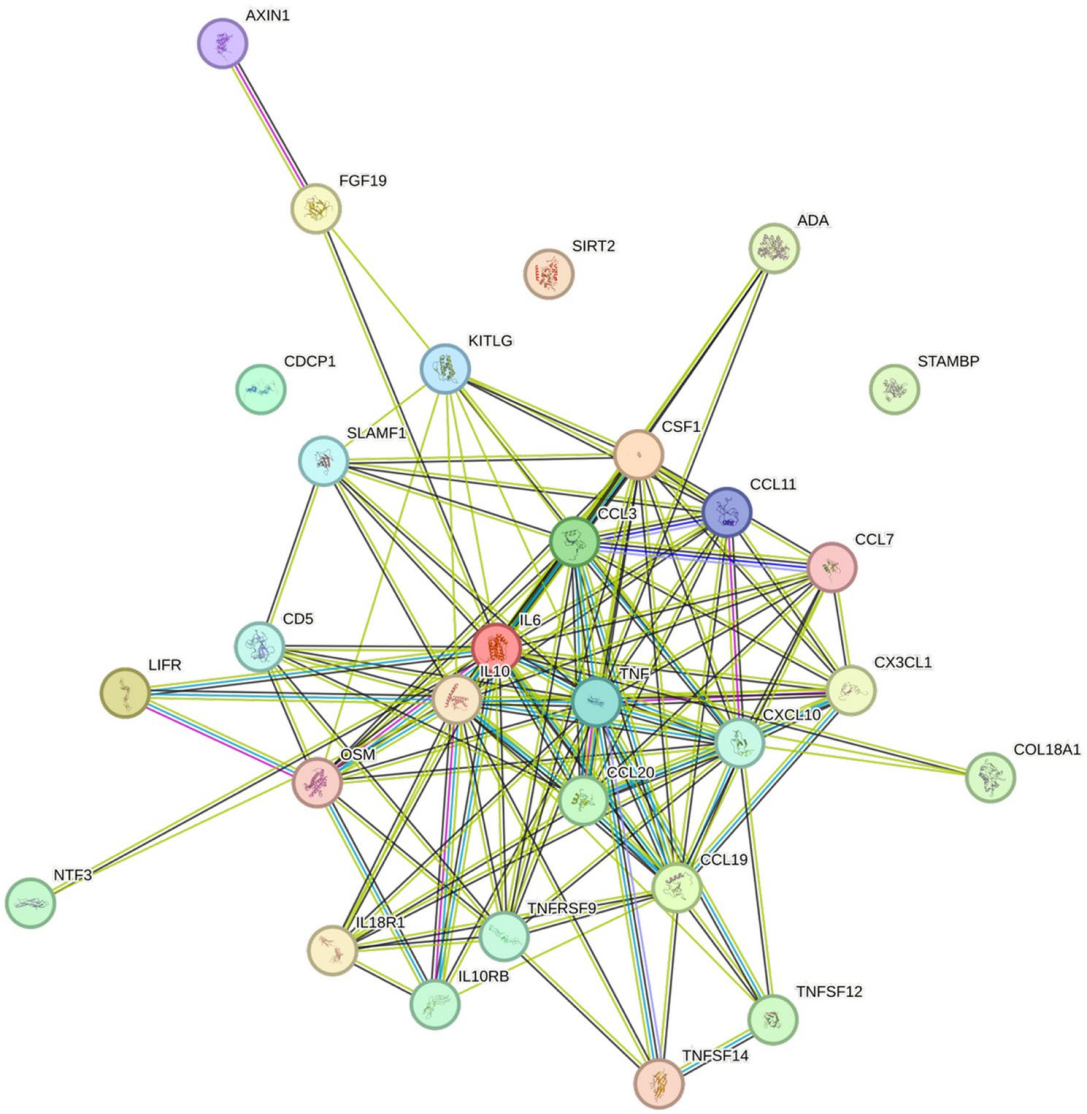
The figure represents a network of interactions among cytokines, demonstrating significant associations between circulating cytokine levels and hsCRP. The nodes are connected by edges of varying thickness, indicating confidence of interactions

Interconnections between CCGFs significantly associated with hsCRP are shown in Fig. 1. As seen, IL6, CSF-1, LIFR, CCL3, CCL19, and TNF are extensively interconnected with other cytokines, whereas CDCP1, SIRT2, and STAMBP do not exhibit any such connections. These cytokines show the strongest associations with hsCRP. However, some cytokines, e.g. CXCL10, CD5, and SLAMF1 displayed several interconnections, without being extensively associated with hsCRP. The STRING network illustrates coordinated cytokine interconnectivity and their associations with hsCRP, highlighting central nodes and high-confidence interactions that suggest biologically coherent inflammatory signaling.

### Correlations between CRP and blood cells

Leucocytes, platelets, and neutrophil cells were weakly correlated with hsCRP (Table 5).

**Table 5:** Correlations in descending order between hsCRP and white blood cells (10^9^/L), platelets (10^9^/L), and neutrophil leucocytes (10^9^/L) in 161 subjects

Blood cells	Spearman	t(N-2)	*p*-value	Benjamini-Hochberg P-values
White blood cells	0.232568	3.0152	0.002990	0.0227
Platelets	0.230591	2.9882	0.003252	0.0227
Neutrophil leucocytes	0.211315	2.7261	0.007127	0.0321

## Discussion

Proatherogenic lipid abnormalities and low-grade systemic inflammation are well-established independent risk factors for cardiovascular events. However, the substantially higher risk observed when both factors coexist suggests parallel yet interacting pathological processes that amplify disease progression [22–24].C-reactive protein (CRP), synthesized in the liver in response to pro-inflammatory cytokines released during tissue injury or infection, functions as an acute-phase protein that enhances complement activation and pathogen clearance, thereby supporting innate immunity ​ [25, 26].

In this study of healthy adults, we demonstrate consistent weak to moderate correlations between blood lipids, hsCRP, a broad panel of cytokines, chemokines and growth factors (CCGFs), and hematologic parameters. These findings reinforce the interconnected nature of low-grade inflammation and lipid metabolism, even in the absence of overt disease, and highlight the potential relevance of hsCRP as an early marker of cardiometabolic risk.

A methodological consideration relates to the use of calculated rather than directly measured LDLC. Although direct LDLC methods are available, they rely on proprietary reagent formulations and differ substantially between manufacturers, limiting their comparability. In Scandinavia, only one large reference material exists for non-HDLC and remnant cholesterol, based on more than 2,600 healthy individuals [27]. In this study, LDLC was estimated using the Friedewald equation [15], which remains the most widely used method in both clinical and epidemiological practice, despite differences from direct LDLC measurement [28]. As our analyses focused on non-parametric correlations, absolute LDLC values were less critical; the rank order of observations—preserved even when estimation introduces small numerical differences—was of primary importance for interpretation.

The possibility of acute inflammation influencing biomarker levels must also be considered. However, this appears unlikely, as active inflammatory conditions are contraindications for mandibular third molar surgery. All participants were assessed by a specialist in maxillofacial surgery (LBE) and showed no clinical signs of inflammation as described classically by Celsus and Galen [29]. Furthermore, the median hsCRP level of 0.7 mg/L supports the absence of clinically meaningful inflammatory activity in the cohort.

The strongest single correlation observed was between hsCRP and IL-6, in line with IL-6’s central role as the primary inducer of hepatic CRP production. This relationship underscores the biological plausibility of hsCRP as a proxy for IL-6-driven systemic inflammation. Significant correlations between hsCRP and additional cytokines—including TNF, IL-10, and CXCL10—reflect a coordinated inflammatory milieu involving both pro- and anti-inflammatory signaling pathways. The relationship with IL-10, commonly viewed as anti-inflammatory, may represent a compensatory response to low-grade immune activation.

Interestingly, our prior work in critically ill COVID-19 patients revealed fewer correlations between CCGFs and hsCRP than those observed here. This contrast likely reflects the dysregulated and heterogeneous cytokine patterns characteristic of severe COVID-19, in which asynchronous elevations of individual cytokines weaken their associations with CRP [30–33]. In healthy individuals, by contrast, inflammatory pathways appear more coordinated and tightly linked.

After FDR correction, 29 CCGFs remained significantly correlated with hsCRP, including CSF-1, CCL3, TNFSF14, and OSM—molecules implicated in monocyte/macrophage activation, endothelial function, and vascular inflammation. These findings suggest that even in ostensibly healthy individuals, subclinical inflammatory signaling engages pathways relevant to atherogenesis and vascular remodeling. Multiplex proteomic profiling thus enables detection of subtle inflammatory patterns that would be missed by single-biomarker approaches.

Consistent with the inflammatory findings, hsCRP was also significantly correlated with several atherogenic lipid parameters—particularly remnant cholesterol, triglycerides (TG), ApoB, and non-HDLC. These lipid species contribute to atherosclerosis not only through cholesterol deposition but also by exerting pro-inflammatory effects that activate endothelial cells and innate immune responses. Remnant lipoproteins, for example, are strongly pro-inflammatory and more atherogenic than LDLC due to their size and lipid composition [34, 35], and have been associated with elevated CRP levels [36]. Elevated TGs correlate with CRP, IL-6, and MCP-1, reflecting chronic low-grade inflammation [37–40], whereas ApoB—representing the total number of atherogenic particles—is a strong predictor of vascular inflammation and cardiovascular risk [41, 42].Non-HDLC provides an integrative measure of atherogenic particles and their inflammatory potential [43, 44], while total cholesterol typically shows weaker associations with inflammation [45, 46].

The correlation between hsCRP and ApoB aligns with previous studies identifying ApoB as a key marker of residual cardiovascular risk. Non-HDLC and total cholesterol also correlated with hsCRP, although to a lesser extent, underscoring the importance of assessing both inflammatory and lipid parameters for comprehensive cardiovascular risk stratification, even in healthy populations.

BMI correlated strongly with hsCRP, consistent with the well-established link between adiposity and systemic inflammation driven by cytokine and adipokine release from adipose tissue. Even modest increases in BMI within the non-obese range can elevate CRP levels, contributing to long-term cardiometabolic risk [47]. We have previously shown that BMI is associated with circulating levels of cytokines and is weakly correlated with white blood cells, neutrophil cells, and platelets [48].

Regarding hematologic markers, hsCRP showed significant associations with leukocyte count, neutrophils, and platelets. These findings suggest that low-grade inflammation is accompanied by subtle changes in immune cell and platelet activity. Neutrophils and platelets are involved in inflammatory signaling and are key players in early atherogenesis, endothelial activation, and thrombotic risk [49–51].

Taken together, our findings demonstrate that in healthy individuals, elevated hsCRP reflects a broader inflammatory and metabolic phenotype characterized by cytokine activation, atherogenic lipid patterns, and hematologic shifts. This supports the growing evidence that subclinical inflammation contributes to early cardiovascular and metabolic risk development. These results also parallel observations from interventional trials such as JUPITER, where individuals with elevated CRP but normal LDLC derived significant benefit from statin therapy, reinforcing the clinical relevance of CRP as an independent risk factor [10].

### Strengths and limitations

A major strength of this study is the use of high‑throughput multiplex proteomics, which enables comprehensive evaluation of a wide range of inflammatory pathways in a relatively large, well‑characterized cohort of healthy adults. The application of robust multiple‑testing correction further strengthens the reliability and interpretability of the findings.

Several limitations should also be acknowledged. First, the generally weak to moderate Spearman’s rank correlations observed between hsCRP and the assessed biomarkers should be interpreted in the context of the study population. Because the cohort consisted of healthy individuals with minimal or no active inflammation, hsCRP values largely fell within the low, subclinical range. Under such physiological conditions, strong correlations with inflammatory biomarkers—typically more pronounced during active inflammation or established atherosclerotic disease—are less likely to occur. Consequently, the correlation strengths observed here may underestimate the magnitude of associations that would be expected in populations with higher inflammatory burden.

Second, the study’s cross‑sectional design limits the ability to infer causality or the directionality of the observed relationships. Although the cohort was healthy, the relatively narrow age range (18–44 years) and limited ethnic diversity may also restrict generalizability to broader populations. Future longitudinal studies, ideally involving more diverse cohorts and a wider range of cardiometabolic risk profiles, are needed to determine whether the biomarker patterns identified here predict the development of future disease.

## Conclusion

In this cross‑sectional study of healthy adults, hsCRP demonstrated meaningful associations with atherogenic lipid measures, circulating CCGFs, BMI, and neutrophil count, revealing a coordinated inflammatory–lipid signature even at subclinical levels. These findings highlight the value of hsCRP as an accessible biomarker that reflects early inflammatory and metabolic processes and may help identify individuals at increased cardiometabolic risk despite the absence of overt disease.

## Supplementary Information

Supplementary Material 1.

Supplementary Material 2.

## Data Availability

The dataset used and analyzed during the current study is available from the corresponding author on reasonable request.
