# Supplementary material for: Significant correlations between blood lipids, cytokines, and C-reactive protein in healthy humans
Source: Lipids Health Dis. 2026 Apr 9;25:107. doi: 10.1186/s12944-026-02939-w (PMC13078040; doi:10.1186/s12944-026-02939-w)
Supplement: Supplementary file 1 — Supplementary Material 1. [file 12944_2026_2939_MOESM1_ESM.zip › 1-20260219_Supplementary Table 1.docx]

| Table 1. |  |  |
| --- | --- | --- |
| **Protein name** | **UniProt ID** | **Gene** |
| Artemin | Q5T4W7 | ARTN |
| Axin-1 | O15169 | AXIN1 |
| C-C motif chemokine 25 | O15444 | CCL25 |
| C-C motif chemokine 28 | Q9NRJ3 | CCL28 |
| CUB domain-containing protein 1 | Q9H5V8 | CDCP1 |
| C-X-C motif chemokine 6 | P80162 | CXCL6 |
| Cystatin D | P28325 | CST5 |
| Delta and Notch-like EGF-related receptor | Q8NFT8 | DNER |
| Eukaryotic translation initiation factor 4E-binding protein 1 (4E-BP1) | Q13541 | EIF4EBP1 |
| Fibroblast growth factor 19 | O95750 | FGF19 |
| Fibroblast growth factor 5 | P12034 | FGF5 |
| Fms-related tyrosine kinase 3 ligand | P49771 | FLT3LG |
| Interleukin-10 receptor subunit alpha | Q13651 | IL10RA |
| Interleukin-10 receptor subunit beta | Q08334 | IL10RB |
| Interleukin-12 subunit beta | P29460 | IL12B |
| Interleukin-15 receptor subunit alpha | Q13261 | IL15RA |
| Interleukin-17A | Q16552 | IL17A |
| Interleukin-17C | Q9P0M4 | IL17C |
| Interleukin-18 receptor 1 | Q13478 | IL18R1 |
| Interleukin-2 receptor subunit beta | P14784 | IL2RB |
| Interleukin-20 | Q9NYY1 | IL20 |
| Interleukin-20 receptor subunit alpha | Q9UHF4 | IL20RA |
| Interleukin-22 receptor subunit alpha-1 | Q8N6P7 | IL22RA1 |
| Interleukin-24 | Q13007 | IL24 |
| Leukemia inhibitory factor | P15018 | LIF |
| Leukemia inhibitory factor receptor | P42702 | LIFR |
| Matrix metalloproteinase-1 | P03956 | MMP1 |
| Matrix metalloproteinase-10 | P09238 | MMP10 |
| Neurotrophin-3 | P20783 | NTF3 |
| Neurturin | Q99748 | NRTN |
| Oncostatin-M | P13725 | OSM |
| Protein S100-A12 (EN-RAGE) | P80511 | S100A12 |
| Signaling lymphocytic activation molecule 1 | Q13291 | SLAMF1 |
| SIR2-like protein 2 | Q8IXJ6 | SIRT2 |
| STAM-binding protein | O95630 | STAMBP |
| Sulfotransferase 1A1 | P50225 | SULT1A1 |
| T-cell surface glycoprotein CD6 | P30203 | CD6 |
| Thymic stromal lymphopoietin | Q969D9 | TSLP |
| TNF-beta (lymphotoxin alpha) | P01374 | LTA |
| TNF-related activation-induced cytokine (TRANCE/RANKL) | O14788 | TNFSF11 |
| Fibroblast growth factor 23 | Q9GZV9 | FGF23 |
| Fibroblast growth factor 21 | Q9NSA1 | FGF21 |
| C-C motif chemokine 3 | P10147 | CCL3 |
| Interleukin-18 | Q14116 | IL18 |
| Stem cell factor | P21583 | KITLG |
| Osteoprotegerin | O00300 | TNFRSF11B |
| Urokinase-type plasminogen activator | P00749 | PLAU |
| Monocyte chemotactic protein 1 | P13500 | CCL2 |
| Adenosine deaminase | P00813 | ADA |
| Caspase-8 | Q14790 | CASP8 |
| C-C motif chemokine 19 | Q99731 | CCL19 |
| C-C motif chemokine 20 | P78556 | CCL20 |
| C-C motif chemokine 23 | P55773 | CCL23 |
| C-C motif chemokine 4 | P13236 | CCL4 |
| CD40L receptor (CD40) | P25942 | CD40 |
| C-X-C motif chemokine 10 | P02778 | CXCL10 |
| C-X-C motif chemokine 11 | O14625 | CXCL11 |
| C-X-C motif chemokine 5 | P42830 | CXCL5 |
| C-X-C motif chemokine 9 | Q07325 | CXCL9 |
| Fractalkine | P78423 | CX3CL1 |
| Interferon gamma | P01579 | IFNG |
| Interleukin-1 alpha | P01583 | IL1A |
| Interleukin-13 | P35225 | IL13 |
| Interleukin-2 | P60568 | IL2 |
| Interleukin-33 | O95760 | IL33 |
| Interleukin-4 | P05112 | IL4 |
| Interleukin-7 | P13232 | IL7 |
| Interleukin-8 | P10145 | CXCL8 |
| Latency-associated peptide TGF-beta-1 | P01137 | TGFB1 |
| Macrophage colony-stimulating factor 1 | P09603 | CSF1 |
| Monocyte chemotactic protein 2 | P80075 | CCL8 |
| Monocyte chemotactic protein 3 | P80098 | CCL7 |
| Monocyte chemotactic protein 4 | Q99616 | CCL13 |
| Natural killer cell receptor 2B4 | Q9BZW8 | CD244 |
| Programmed cell death 1 ligand 1 | Q9NZQ7 | CD274 |
| T-cell surface glycoprotein CD5 | P06127 | CD5 |
| T-cell surface glycoprotein CD8 alpha | P01732 | CD8A |
| Tumor necrosis factor ligand superfamily member 12 (TWEAK) | O43508 | TNFSF12 |
| Tumor necrosis factor | P01375 | TNF |
| Tumor necrosis factor ligand superfamily member 14 | O43557 | TNFSF14 |
| Tumor necrosis factor receptor superfamily member 9 | Q07011 | TNFRSF9 |
| Interleukin-10 | P22301 | IL10 |
| Interleukin-5 | P05113 | IL5 |
| Eotaxin | P51671 | CCL11 |
| Beta-nerve growth factor | P01138 | NGF |
| Glial cell line-derived neurotrophic factor | P39905 | GDNF |
| Transforming growth factor alpha | P01135 | TGFA |
| TNF-related apoptosis-inducing ligand | P50591 | TNFSF10 |
| Interleukin-6 | P05231 | IL6 |
| Hepatocyte growth factor | P14210 | HGF |
| Vascular endothelial growth factor A | P15692 | VEGFA |
| C-X-C motif chemokine 1 | P09341 | CXCL1 |
| Table 1. The Proseek Multiplex Inflammation panel contains 92 inflammation-related proteins, here presented with protein name, defined UniProt ID and gene symbol. | | |
